# Giant Cell Tumor of the Frontal Bone: A Rare Case Report and Review of Literature

**DOI:** 10.7759/cureus.52834

**Published:** 2024-01-23

**Authors:** Mohammed AR Abdellatif, Karam Rabi, Ahmed t Ghanem, Ahmed Dawoud, Izzeddin A Bakri

**Affiliations:** 1 Department of Medicine, Faculty of Medicine and Health Sciences, An-Najah National University, Nablus, PSE; 2 Department of Neurosurgery, Palestinian Medical Complex, Ramallah, PSE; 3 Department of Pathology, Al-Makassed Islamic Charitable Hospital, Jerusalem, PSE

**Keywords:** giant cell tumor of bone, new technologies in neurosurgery, neurosurgery, skull, frontal headache, brain ct scan

## Abstract

Giant cell tumors (GCTs), typically benign, predominantly manifest in individuals aged 20-40, with the most common locations being the metaphysis or epiphysis of the femur or tibia. Infrequently, they may occur in the skull. Despite their benign nature, these tumors can exhibit aggressive behavior and have the potential to metastasize. In the case at hand, a 20-year-old female presented to the hospital with a progressively enlarging right frontal swelling over the preceding months. The patient reported intermittent headaches, alleviated by analgesics, and exhibited a normal neurological examination along with a Glasgow Coma Scale (GCS) score of 15 out of 15. Imaging revealed an expansive soft tissue mass in the right frontal bone involving both inner and outer tables. Surgical intervention was pursued through a right frontal incision followed by tumor excision. Histopathological examination of the specimen confirmed the presence of a GCT. The limited existing literature on this topic highlights the need for further research and insights into effective strategies. This case contributes to addressing this gap in knowledge, offering valuable information to enhance our understanding of the challenges associated with similar rare cases and improve patient outcomes.

## Introduction

Giant cell tumor (GCT) of bone is a primary, benign, yet locally aggressive bone tumor characterized by significant tissue destruction at the epiphysis of long bones. Typically seen in young adults aged 20 to 40, it has a slight female predilection and constitutes about 5% of all bone tumors and 20% of benign bone tumors [1-3]. The primary sites of occurrence are the distal femur and proximal tibia in nearly 50% of cases, with potential involvement in the skull and pelvis [4,5]. In a study comprising 110 cases of GCT in the skull, findings revealed predominant locations as follows: temporal involvement in 37 patients, sphenoid in 20 patients, occipital in six patients, frontal in two patients, and the temporomandibular joint in two patients [6].

While GCT mainly affects the axial skeleton, including the skull, mandible, and pelvis, especially in cases of GCT/Paget’s disease of bone (GCT/PDB), notable differences exist in terms of frequency, age onset (20-40 years in GCT and >40 years in GCT/PDB), and skeletal localization. GCTs associated with Paget's disease are more likely to present with multiple lesions [7,8]. A hereditary component, particularly in cases affecting the skull and pelvis associated with Paget's disease, has been noted. Recent research has unveiled distinct genetic backgrounds between isolated GCT and GCT associated with Paget's disease, leading to unique biochemical and histological characteristics [7].

Metastases occur in (1-9%) of GCT patients, primarily observed in the lungs, constituting the most common secondary site. Sporadic case reports also document occurrences in the lymph nodes, bone, skin, and breast [9,10]. In addition, there have been documented instances of malignant transformation of GCT [11]. Clinical symptoms, including pain, local swelling, and limited joint motion, are nonspecific and related to the affected bone [12]. Computed tomography (CT) and magnetic resonance imaging (MRI) are standards for evaluating GCT. Radiographically, GCT exhibits a characteristic radiolucent, geographic appearance with a narrow transition zone at the lesion's margin. The histological appearance includes abundant giant cells with a benign epithelioid to spindle-shaped mononuclear cell background. The histologic grading system has a limited clinical value in predicting tumor behavior [9,12].

Surgical treatment remains the preferred choice for GCT. Depending on the articular surface involvement, the tumor can be removed via resection or curettage, with or without local adjuvants. Optimal outcomes are achieved when the tumor is removed with tumor-free margins, minimizing surgical morbidity and ensuring an acceptable functional outcome. Resection with wide (microscopically negative) margins has been associated with few or no recurrences ranging from 0% to 16%, albeit with a poorer functional outcome and increased surgical morbidity [12].

## Case presentation

Patient presentation

**Figure 1:**
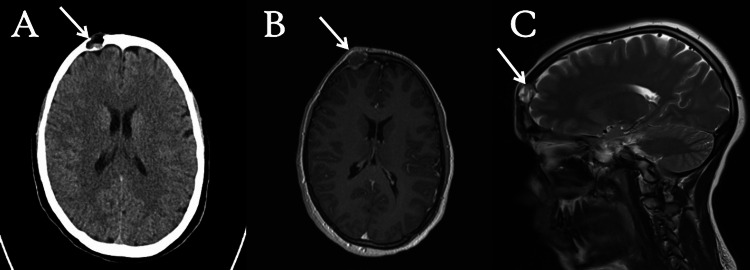
Preoperative imaging including MRI and CT scan. (A) CT scan without contrast shows an expansive soft tissue mass with bevelled edges, measuring approximately 1.5 x 1.8cm in the right frontal bone. Notably, the mass reaches the surface of dura without infiltrating the brain parenchyma (white arrow). (B) T1-weighted MRI axial view reveals a hypo-intense lesion on the right frontal skull lesion (white arrow). (C) T2-weighted MRI sagittal view reveals tumor impingement of the dura (white arrow). CT: computed tomography; MRI: magnetic resonance imaging

A 20-year-old patient, with an unremarkable family history, presented to the hospital with an eight-month history of swelling on the right forehead, which has gradually increased to approximately 2 x 2 cm. The patient also reported experiencing intermittent headaches over the past several months, alleviated by analgesia. Upon examination, the swelling was localized to the right forehead, with no associated lymph node enlargement and normal neck mobility. On neurological examination, the patient exhibited a Glasgow Coma Scale (GCS) score of 15/15, normal pupil reactions, and regular tone, reflexes, and power in both upper and lower limbs. MRI and non-contrast CT scans of the head were conducted, as shown in Figure 1, to rule out the presence of other suspected pathologies, such as osteosarcoma, fibrous dysplasia, and cystic lesions.

Surgical operation technique

The patient was positioned in a supine posture, and a precise horizontal skin incision was made approximately 10 cm behind the hairline. Subsequently, the flap was carefully retracted until the bony lesion was fully exposed. The bony lesion was excised until the visualization of a normal bone and dura was achieved and specimens were taken for histopathological purposes. The resultant bony defect was addressed by applying bone cement, secured in place with sutures. Following this, the subcutaneous layer was diligently closed, and the skin was sealed with a stapler. The operation proceeded smoothly without any intra- or post-operative complications. The patient was discharged the following day with instructions provided for wound care, and arrangements were made for a follow-up appointment in two weeks.

Histopathology report

**Figure 2:**
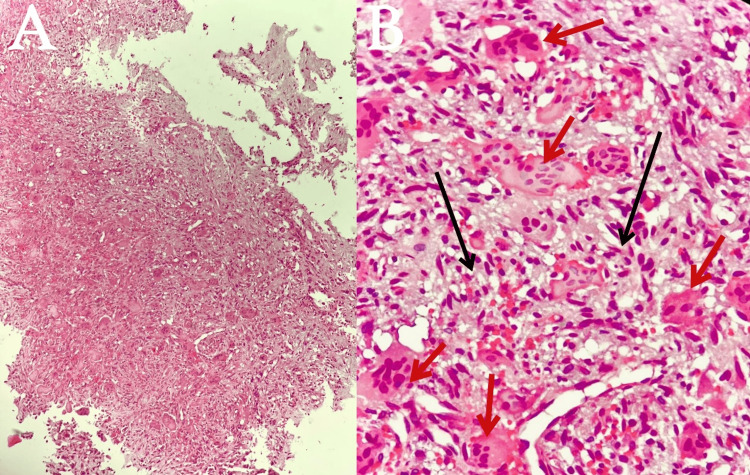
Microscopic views of the pathology specimen captured at varying levels of magnification. (A) H&E: 10x, (B) H&E: 40x Cellular lesions composed of a large number of osteoclast-like giant cells (red arrows), between which mononuclear cells are embedded (black arrows). No atypical mitosis or necrosis seen. H&E, hematoxylin and eosin

The submitted specimen consisted of fragments of pink fibromembranous and bony tissue, with an aggregate measurement of 1.5 x 1.5 cm. Histological examination revealed a cellular lesion characterized by a substantial presence of osteoclast-like giant cells. Interspersed between these giant cells were mononuclear round to oval cells and spindled cells exhibiting a pale eosinophilic cytoplasm, nuclei with dispersed chromatin, and small nucleoli, as illustrated in Figure 2. No atypical mitotic figures were observed, and no evidence of necrosis was identified. Common features included aneurysmal changes, clusters of hemosiderophages, and fibrotic areas. Focal destruction of the cortical bone was evident, replaced by a reactive rim of a woven bone at the lesion's periphery.

## Discussion

The case of a GCT located in the frontal bone significantly enhances our understanding of the clinical, surgical, and histopathological aspects of this rare skull-based tumor. This discussion will delve into key aspects of GCT, including genetic variations, metastatic tendencies, clinical complications, surgical intricacies, and histopathological findings. The documented instances of metastasis, ranging from 1% to 9%) among GCT patients and predominantly affecting the lungs, emphasize the need for vigilant follow-up and systematic imaging surveillance [13]. Furthermore, the infrequent occurrences of malignant transformation within GCT introduce considerations that require careful management.

GCTs typically manifest as benign yet locally aggressive lesions with the capability of metastasis. This report adds to the limited literature available on these skull tumors. Pain and swelling commonly characterize their presentation. Radiographically, they exhibit as radiolucent lesions lacking sclerotic borders, frequently observed in the sphenoid bone [14]. The clinical manifestation, marked by a gradual swelling of the forehead and intermittent headaches, mirrors the nonspecific symptoms commonly associated with GCT. However, the complexity in this case arises from the tumor's unique location in the frontal bone, highlighting the necessity for heightened clinical insight in less prevalent anatomical sites [15]. Histopathological analysis provides profound insights into the distinctive features of the GCT, notably the presence of osteoclast-like giant cells, mononuclear round to oval cells, and spindled cells. These observations align harmoniously with the recognized histological attributes typically associated with GCT [16].

In a documented rare case from 2018, a GCT of the frontal bone was identified. Subsequent investigations and management underscored the limited predictive value of both radiographic and histopathological findings for clinical outcomes in such instances. It was clearly evident in this case report, which demonstrated the pivotal role of early surgical intervention as the definitive predictor of disease prognosis, ensuring optimal patient outcomes and minimizing the risk of future complications [7].

## Conclusions

GCTs, typically considered benign, present unique challenges when situated in the skull, given their potential for aggressive behavior. In this case report, thorough investigations revealed the presence of a GCT of the frontal bone. The patient underwent surgical intervention, which was executed successfully without complications. Considering the limited existing literature on GCTs of the skull, particularly of the frontal bone, documenting cases like this one is vital for expanding our understanding of these rare occurrences. Contributing such clinical insights can play a significant role in advancing medical knowledge and guiding future approaches to diagnosis and management.
